# Bioethics and the value of disagreement

**DOI:** 10.1136/jme-2024-110174

**Published:** 2024-08-31

**Authors:** Michael J Parker

**Affiliations:** 1 The Ethox Centre, Oxford Population Health, University of Oxford, Oxford, UK

**Keywords:** cultural diversity, ethics

## Abstract

What does it mean to be a bioethicist? How should the role(s) of bioethics be understood in the context of a world of intense value conflict and polarisation? Bioethics is—in all its various forms and traditions—potentially well-positioned to contribute to addressing many of the most pressing challenges of value polarisation and conflict in diverse societies. However, realising this potential is going to require moving beyond currently foregrounded methods and developing new models for engaging with moral disagreement. This paper proposes an approach, ‘adversarial cooperation,’ drawing on the concepts of ‘adversarial collaboration’ from the sciences and ‘antagonistic cooperation’ from the humanities. Adversarial cooperation aims to combine the rigour and structured methodology of adversarial collaboration with the cultural sensitivity and expansive vision of antagonistic cooperation. The paper also addresses key challenges to adversarial cooperation, including ethical considerations, tensions between substantive and procedural values, the problem of misinformation and the need for decision-making amidst ongoing disagreement. Ultimately, adversarial cooperation suggests a reimagining of bioethical expertise, emphasising skills in mediation, the arts and humanities and participatory decision-making alongside established philosophical competencies. This implies a model of normative bioethical authority grounded in the ability to facilitate inclusive and trustworthy processes of moral deliberation. Realising the potential of adversarial cooperation will require significant changes in bioethics training and practice, as well as a commitment to reflexivity, humility and the amplification of marginalised voices. By embracing this approach, bioethics can play a vital role in navigating the complex moral landscapes of pluralistic societies.

 My work with the Global Health Bioethics Network has been an important ongoing reminder that bioethics means different things in different places and is practised in very many different ways.^1^ Partly despite this and to a large extent because of it, bioethics is uniquely positioned to contribute to addressing the pressing challenges posed by value polarisation and conflict in increasingly complex, diverse and interconnected societies. As we grapple with issues ranging from pandemic response and climate change to the deployment of new technologies such as AI, and enduring ethical problems such as those relating to the end of life, reproductive medicine, and resource allocation, the need for effective strategies to navigate deep differences in values and worldviews has never been more urgent.^2^ All too often, these value conflicts lead to entrenched divisions, mutual incomprehension and a breakdown of social cooperation, undermining our ability to find common ground and take collective action on critical issues. Bioethics has the potential to play a vital role in bridging these divides and fostering productive dialogue across difference. To realise this potential, however, the field must move beyond currently foregrounded methods and frameworks and develop new models for engaging with the full complexity of moral disagreement in the context of pluralism. In this paper I suggest that an integration of two concepts arising from different intellectual traditions—‘adversarial collaboration’ from the sciences and ‘antagonistic cooperation’ from the humanities—offers valuable resources for engaging bioethical polarisation. Arising from different contexts, these approaches share a recognition of the inevitability and even productivity of disagreement, and a commitment to channelling it towards constructive ends through structured engagement across difference.

After briefly reviewing the two concepts and their origins, I argue that their respective strengths and limitations suggest the value of an integrative approach, which I term ‘adversarial cooperation.’ I outline a strategy for implementing this approach and go on to describe challenges any successful and coherent implementation will need to navigate. Throughout, I highlight the special value of the arts and humanities in engaging effectively with value conflicts.

## Adversarial collaboration: resolving scientific disputes

There is currently a great deal of excitement in scientific circles about the potential of ‘adversarial collaboration’ as a method for resolving important empirical and theoretical disagreements between scientists.^3 4^ The use of adversarial collaboration was pioneered by Daniel Kahneman.^5^,^7^ A Nobel laureate known for his work on cognitive biases and decision-making,^8^ Kahneman was motivated to adopt the approach because of his frustration with unproductive debates in psychology and other fields. He observed that scholars with opposing views often talked past each other, selectively citing evidence that supported their positions while ignoring or dismissing contradictory findings.^9^ Kahneman believed a more collaborative and systematic approach to resolving empirical disputes could help advance scientific understanding and build trust among researchers.

In an adversarial collaboration, scholars who hold opposing views on a scientific question agree to work together to design and conduct a joint study that can adjudicate between their hypotheses. Key steps include: (1) identifying the precise points of disagreement; (2) agreeing on what evidence would resolve the dispute; (3) collaborating to gather that evidence; and (4) publishing the results jointly,^1^ regardless of whose hypothesis is supported.^5 6^ By requiring researchers to clarify their predictions and subject them to rigorous tests, adversarial collaborations have the potential to generate high-quality evidence that moves debates forward. Adversarial collaborations have been deployed to resolve debates in a range of domains, including psychology and neuroscience. In psychology, for example. the approach has been used to study topics such as gender bias in science^10^ and dual-task costs in working memory.^11^ In neuroscience, adversarial collaborations have tested competing theories of consciousness. ^12^

Adversarial collaboration has important strengths. It forces scholars to clarify their disagreements and confront them head-on, rather than talking past each other. It subjects contested claims to rigorous tests, generating evidence that can move debates forward.^5 6^ Perhaps most importantly, it embodies a spirit of good faith and mutual respect, even in the face of deep disagreement.^13^ By working together to design and conduct joint studies, researchers who disagree can build understanding and identify areas of common ground. It is noteworthy that there have been some calls to develop approaches to adversarial collaboration to engage with the social and ethical dimensions of science.^14^ ^15^ Notwithstanding its success in the scientific context, however, adversarial collaboration has important limitations as a candidate methodology for engaging effectively with value disagreements. Developed to resolve empirical disputes between scientific experts, it is likely to struggle to accommodate the entanglement of facts and values, diversity of lived experiences and power dynamics that characterise many bioethical controversies.^16 17^ Its emphasis on experimental evidence underplays the importance of narrative, emotion and identity in moral life.^18 19^ By privileging scientific expertise, it risks marginalising vital perspectives from the humanities, social sciences and affected communities.^20^ This suggests that while adversarial collaboration can be a useful tool for testing empirical claims, engaging the full complexity of value conflict and moral disagreement requires a more inclusive and multidisciplinary approach.^21^

## Antagonistic cooperation: cultural tensions as creative resource

The concept of ‘antagonistic cooperation’ has very different origins. It arises from a strand of African-American thought that finds its clearest expression in the writings of Ralph Ellison, Albert Murray and more recently, Robert O’Meally.^22^ There is some uncertainty about the origins of this use of the concept.^22^ However, it was Ellison, in his essays and correspondence who first used the term to describe the way distinctively American cultural forms like jazz emerge from the tension and interplay between diverse, even oppositional cultural elements—African and European, high and low sacred and profane.^23^ Ellison stresses how much American ‘newness’ comes from creatively repurposing and recombining old cultural elements and suggests that polarisation might be seen as a difficult but inescapable part of the creative process by which a democratic society metabolises new challenges and ideas. With antagonistic cooperation, unexpected influences and innovative remixing of cultural elements, new shared understandings can emerge from the parts. Albert Murray extended this idea, arguing that apparently antagonistic cultural forces can cooperate to produce novel syntheses transcending their separate elements. Murray argues that American culture has been shaped by a process of antagonistic cooperation between black and white Americans. He suggests that this process, while often fraught and painful, has also been a source of immense creativity and dynamism in American life.^24^

At the heart of antagonistic cooperation is an agonistic vision of diversity and disagreement as potential creative resources, not just obstacles to be overcome. It assumes an irreducible cultural pluralism but sees this as a source of social and artistic innovation where diverse elements can be brought into productive interaction within a shared democratic framework. Crucially, this productive pluralism is understood as emerging organically from lived experience and practical activity, not abstract theories. As a sensibility forged in a context of cultural marginalisation and oppression, antagonistic cooperation is attuned to the power dynamics often elided in scientific discourses. It insists on engaging the full texture of moral experience in all its ambivalence and particularity. And it locates possibilities for cooperation and new understanding in concrete practices, not just explicit dialogue—in how we make music, tell stories or organise communities.

Rooted in cultural theory and aesthetics, antagonistic cooperation offers a rich vision of pluralism’s promise but little concrete guidance for policy or practice. It has limitations, therefore, as a practical framework for engaging bioethical controversies and value conflict head-on. Its emphasis on organic interaction rather than structured deliberation makes it difficult to apply in formal decision-making contexts. And its focus on cultural expression can miss some of the institutionally entrenched obstacles to cooperative engagement across difference.

## Adversarial cooperation: an integrative approach

Adversarial collaboration and antagonistic cooperation offer productive but different strategies for understanding the dynamics of conflict and collaboration in various contexts, including the polarised landscapes of contemporary societies. There are important differences between these ideas. Adversarial collaboration arises from behavioural science and psychology, while antagonistic cooperation emerges from a context of cultural studies and literary criticism, particularly in relation to African-American experience. Adversarial collaboration is focused on structured, intentional collaborations between researchers or thinkers with opposing views, while antagonistic cooperation refers to broader social and cultural processes of contestation and exchange. Adversarial collaboration places emphasis on the collaborative aspect, framing disagreement as a tool for rigorous and reliable inquiry, while antagonistic cooperation has a more agonistic or conflict-oriented connotation, suggesting that the process of cooperation is inherently marked by tension and struggle. Adversarial collaboration tends to be more focused on the pursuit of truth and the disciplined resolution of intellectual disputes, while antagonistic cooperation is associated with the creative and dynamic emergence of new cultural forms and identities.

Notwithstanding these differences, there are important points of connection. Both concepts recognise the inherent presence of conflict and disagreement in human interactions and systems. They suggest that opposing perspectives or forces, when engaged productively, can lead to new insights, creativity and progress. Both emphasise the importance of forging respect and a degree of open-mindedness towards opponents or intellectual adversaries. And both challenge the notion that disagreement is inherently negative or unproductive, instead pointing to its potential benefits. The complementary strengths and weaknesses of adversarial collaboration and antagonistic cooperation suggest the potential value of an approach combining their insights. Such an integration—which I have chosen here to call ‘adversarial cooperation’—would unite the rigour and structured methodology of the former with the cultural sensitivity, arts and humanities focus and expansive vision of the latter. Fully engaging these resources will require bioethicists to venture beyond disciplinary comfort zones—but the potential rewards are significant.

An approach to polarisation and value conflict drawing on these traditions would emphasise the following. First, it would pay particular attention to the development of effective ways of convening opposed perspectives to explore and articulate key disagreements, not just in empirical beliefs but also in underlying values, commitments and lived experiences. In so doing, it will draw on and fully integrate the humanities, arts and other creative practices to enable mutual understanding across worldviews, recognising antagonistic cooperation’s emphasis on creativity and agonism. Second, it would require the cooperative design of and commitment to a structured process agreed by all to have the potential to be transformative of current positions. This will involve the collaborative definition of evaluative criteria reflecting the plurality of relevant values—combining adversarial collaboration’s requirement for discipline with antagonistic cooperation’s pluralism and experiential sensitivity. Third, it would require agreement on and commitment to two important forms of coproduced outcome. The first is to coauthor a paper (or other relevant pre-agreed output) irrespective of the outcome. The second, reflecting the normative purpose, is to collectively undertake or recommend at least one morally significant action—remembering, of course, that a decision to do nothing can also sometimes be a moral significant act. Together these features of adversarial cooperation capture the idea of a structured, iterative method for navigating complex ethical terrain through careful, inclusive, incremental steps towards greater mutual understanding and more ethically robust positions

## Sketch for a process

It will be apparent from the above that a process meeting these requirements might take a range of different forms. This is an important advantage of this account because it suggests a process capable of being rigorous, culturally sensitive and coproduced in ways that are adequate to the task of engaging effectively with value conflict in different contexts. The requirements outlined above do, however, suggest the following general features might be characteristic of an adversarial cooperation approach.

### Setting the stage and coproducing the process

An initial phase will involve selection and preliminary characterisation of the value conflict to be explored, and identification of a relevantly diverse range of perspectives, forms of expertise and experience. It will likely also require the appointment of a trusted facilitator who will instigate relevant and culturally appropriate activities to foster empathy and mutual understanding. An essential part of this first phase will be addressing the second and third requirements described above that is, the cooperative design of and formal commitment to a structured process with the potential to be transformative, the collaborative definition of criteria for evaluation and agreement on and commitment to the two required coproduced outcomes—a coauthored publication and agreement to undertake, and evaluate at least one normatively significant collective action as a consequence of the process.

### Value topography exploration

The precise methodology to be adopted will be shaped by the outcome of phase I. However, it is likely that this will take the form of a facilitated collaborative exploration of the value topography of the problem under investigation comprising three main interconnected and overlapping tasks. One will be a *comprehensive value mapping* in which the moral values at stake will be elucidated and clarified. This may involve input by humanities scholars and social scientists to provide historical–cultural context, and use of artistic methods to visualise the values landscape—from which new shared understandings or awareness of previously unexpected overlaps may emerge. A complementary task will be *factual topography assessment* in which agreed and contested factual claims are identified collaboratively. Making progress here will involve consulting diverse sources (see ‘Fake news and misinformation’ section below for more discussion on this). In some cases, where contested factual claims are amenable to testing, targeted ‘adversarial collaboration’ along the lines advocated by Kahneman *et al* might be considered. A third task will involve *delineating areas of agreement and persistent disagreement*, mapping out relevant moral arguments, and potentially engaging in activities to adjust ethical positioning and establish some new areas of consensus. The goal here is not necessarily to reach full consensus—though this may be welcomed where it emerges—but rather to deepen understanding, clarify differences, identify any areas of overlapping consensus and explore possibilities for cooperation or compromise. As in previous tasks, the use of creative methods may usefully enable further refinement and understanding of the ethical terrain.

### Next normative step formulation

Following and building on the exploration described above, phase III will involve a shift in focus to the codesign of possible practical ethical steps towards achieving a more ethically robust position. This facilitated process, which will in many cases be contentious and will always depend on goodwill and mutual respect on all sides, will likely include consideration of historical and cross-cultural examples and the use of creative methods to identify potential paths and ways of acknowledging trade-offs involved in the different available options. The endpoint of this phase will be agreement of a normatively significant step and of requirements for its evaluation. As mentioned above, a normatively significant step might range from a minor policy adjustment or shift in public engagement all the way through to changes or policy interventions of a much more significant kind. It might also, on occasion, involve a choice to refrain from action. The requirement is that it is agreed to constitute a step towards a more ethically robust position.

### Implementation, reflection and evaluation

The final phase of each iteration of the process will be the practical implementation of the step and its observation, documentation, and evaluation according to pre-agreed criteria across the various ethically significant dimensions identified in the previous phase. In all cases, the process will involve the completion of pre-agreed tasks such as coauthoring of a paper or other pre-agreed output reflecting on the ethical path traversed, capturing enduring value disagreements that is, the value topography, and noting any shifts in position. Future iterations of the adversarial cooperation process may be undertaken as needed.

The approach outlined here frames the engagement with moral value conflicts as a continuous, iterative process of ethical improvement. It emphasises the importance of careful, incremental progress while maintaining flexibility and openness to different perspectives. By conceptualising the process in this way, a structured yet adaptive framework is provided for navigating complex ethical terrain, suitable for both theoretical exploration and practical decision-making in pluralistic contexts. A helpful feature of this model is that it does not imply an expectation that the process will, or even ought to, reach a point at which there is no longer significant value difference or disagreement. Indeed, antagonistic cooperation, with its emphasis of the creative and dynamic emergence of new cultural forms and identities, suggests that this would often be undesirable as an outcome. Neither does it assume that there will not be setbacks such as periodic losses of confidence among participants in a process where this does not yield immediate, dramatic changes. To address these challenges, the process will need to include regular reflexive pauses to reassess the broader ethical landscape and long-term goals and will need to maintain open communication with all stakeholders about the nature and intent of this gradual approach.

## Some challenges adversarial cooperation will need to address

This sketch suggests that adversarial cooperation holds promise as a methodology for navigating value conflict. But much work remains to be done to refine its theoretical foundations and evaluate its practical utility across varied domains. Approaches would need to be adapted and tailored to the specific context and goals at hand and would require careful facilitation, design and evaluation to ensure that they are inclusive, equitable and productive, and that power dynamics are addressed. While adversarial cooperation has potential as a useful method in practical bioethics, it also faces at least four important challenges. Some of these are to do with ethical questions likely to arise in its practical implementation. Others arise out of tensions between substantive and procedural considerations. Some of them concern how to deal with the pervasive nature of misinformation. Still others relate to the tensions between the commitment to open dialogue and the need to make decisions.

### Ethical considerations in the uses of adversarial cooperation

Any model of adversarial cooperation capable of commanding well-founded trust and confidence will need to be grounded in ethical values and commitments ensuring that it remains true to the spirit of good-faith engagement and mutual respect that underlies both adversarial collaboration and antagonistic cooperation. The ability of adversarial cooperation approaches to productively mediate polarisation and value conflict will hinge on paying careful attention to power dynamics, inclusivity and cultural difference. It will be vital to ensure that historically marginalised voices are centred, not drowned out by dominant scientific and bioethical discourses.^25^ Participants will need to represent the range of affected communities, not just credentialed experts. It will be important for it to recognise that not all participants will have equal access to platforms, resources or institutional support, and that these imbalances can shape the terms and outcomes of dialogue in ways that may reinforce rather than challenge existing inequities. It will be vital to actively work to level the playing field and amplify marginalised voices, so that the benefits of productive disagreement are not reserved only for the privileged. The impacts of proposed interventions on disparately situated groups will need to be rigorously assessed to guard against unintended harms.

Another key ethical consideration is the need to balance the value of productive disagreement with the importance of social cohesion and mutual understanding. While methods of adversarial cooperation can lead to new insights and progress, they also risk exacerbating divisions and eroding trust if not approached with care. It is crucial to ensure that these practices are guided by a genuine commitment to good-faith dialogue and intellectual humility rather than devolving into bad faith point-scoring or entrenched factionalism. This means that those engaging in these forms of dialogue have a moral obligation to strive for empathy and to truly seek to understand the perspectives and motivations of those they disagree with. They must be willing to challenge their own assumptions and biases, and to remain open to the possibility of changing their minds in the light of new evidence or arguments—epistemic humility. At the same time, they must be able to robustly advocate for their own positions and values, while maintaining respect for their intellectual opponents as human beings deserving of dignity and consideration.

Relatedly, it is going to be important to consider the potential unintended consequences of adversarial cooperation in the realm of moral and social debate. While these approaches can help to surface new perspectives and challenge entrenched positions, they may also risk giving undue legitimacy to harmful views, or contributing to a climate of relativism and uncertainty that undermines the pursuit of ethical progress. Those engaging in these practices have a responsibility to exercise judgement and discretion in deciding which perspectives are worthy of serious engagement, and to develop respectful ways of identifying and discussing ideas that are fundamentally incompatible with the success of adversarial cooperation. Ongoing self-reflection and adjustment in response to critical feedback will be essential. I discuss this further in the Fake news and misinformation section below.

### Tensions between substantive and procedural values

Ultimately, the key to ethically deploying these methods in the context of value conflict may lie in maintaining a careful balance between intellectual openness and moral conviction. This is no easy task. It requires ongoing reflection, humility and a willingness to grapple with difficult tensions and trade-offs. This points to an inherent tension at the heart of any model of adversarial cooperation. On one hand, adversarial cooperation is premised on the idea of participants engaging openly and in good faith with perspectives that differ from their own and being willing to subject their own beliefs and assumptions to critical scrutiny. This requires a degree of intellectual humility and flexibility, and a willingness to entertain the possibility that one’s own views may be wrong or incomplete. On the other hand, its success depends on certain core values and commitments. The question, then, is how to reconcile the openness and flexibility demanded by adversarial cooperation with a commitment to certain non-negotiable principles essential to its meaningful functioning. If we hold too tightly to our core convictions or if the set is too expansive, we risk closing ourselves off to the very possibility of productive engagement with those who see things differently. But if we are too quick to compromise on our fundamental values, or if the set is too small, we risk losing the moral and intellectual integrity of the process.

There is no easy answer to this dilemma, and different practitioners may strike the balance in different ways.^26^ One possible approach is to distinguish between the substance of core principles and the specific ways in which those principles are interpreted and applied in practice. In other words, we might remain firmly committed to certain non-negotiable values such as respect for persons and epistemic humility while recognising that there can be legitimate disagreement and debate about how these values should be understood and put into practice in particular contexts. This allows for a degree of openness and flexibility in the realm of policy and implementation, while still maintaining a strong foundation of shared moral and intellectual commitments. Another approach is to view adversarial cooperation not as a means of resolving or eliminating fundamental value differences, but rather as a way of managing and navigating those differences in a constructive way.^2^ The goal, on this view, is not to arrive at a final consensus or to persuade others to abandon their core convictions, but rather to find ways of coexisting and working together despite our disagreements. This might involve seeking out areas of common ground or overlapping consensus where possible, while also acknowledging and respecting the ways in which our fundamental beliefs and values may diverge. It means being willing to engage in ongoing dialogue and negotiation, while also maintaining the integrity of our own moral and intellectual commitments.

Ultimately, the tension between and interdependence of adversarial cooperation and non-negotiable values is a real and ongoing one that requires careful navigation and constant balancing, and a willingness by all parties to live with a degree of uncertainty and discomfort. But by approaching this tension with honesty, good faith and a deep sense of ethical responsibility, we can strive to create spaces and practices of engagement that allow for productive disagreement and collaboration across difference.

### Fake news and misinformation

A particularly intense contemporary problem for the successful implementation of any version of adversarial cooperation arises out of the pervasiveness of misinformation and disinformation.^27^,^30^ While the approach emphasises the importance of treating opponents as legitimate adversaries, it also presumes a certain baseline of shared factual reality and commitment to good-faith argumentation. When one or more sides of a debate are operating from false beliefs or deliberately spreading misinformation, this has the potential to undermine the conditions for productive agonistic contestation. Advocates for adversarial cooperation will likely be strongly supportive of efforts to create a healthier epistemic environment in which the contestation necessary for adversarial cooperation can productively occur. This might include a range of measures to improve the information ecosystem and combat misinformation, such as funding for public media, support for fact-checking organisations or regulations on social media platforms. However, the problem of misinformation is not likely to be addressed in the short term.

Against this background, advocates of adversarial cooperation would emphasise the importance of continually seeking ways to engage across differences and find productive paths forward, even in the face of serious epistemic divides. This may require difficult judgement calls and a willingness to set boundaries against truly bad faith actors. But the underlying commitment to pluralism, coexistence and the transformative potential of conflict should remain. The goal is to create the conditions under which passionate but constructive contestation is possible, even if this is an ongoing struggle in an age of misinformation. There are likely to be many different ways in which adversarial cooperation might navigate this tension. These may include: (1) distinguishing between good-faith and bad-faith actors—not everyone who believes or spreads false information is doing so maliciously. Some may simply be misinformed or caught up in epistemic bubbles. An adversarial cooperation approach could emphasise the importance of engaging with good-faith actors to try to establish a shared factual basis, while being more willing to marginalise or exclude bad-faith actors who knowingly spread disinformation. (2) Focusing on the identification and development of shared underlying values and concerns: even when people hold demonstrably false beliefs, there are often real values, concerns and experiences underlying those beliefs.^31^ An adversarial cooperation approach could focus on engaging with and validating those underlying sentiments while firmly decoupling them from the false beliefs. This could involve affirming the legitimacy of someone’s fear or sense of unfairness, for example, while challenging the specific misinformation they call on. (3) Having explicit conversations about what the criteria ought to be for legitimate expertise and reliable sources of information with the aim of both understanding the other’s conception of knowledge and expertise and also of potentially identifying a shared subset of sources.

### The need to make decisions and develop policy

There are important practical challenges—and challenges of credibility—in implementing adversarial cooperation in real-world contexts where decisions must be made and policies enacted, often under significant time and resource constraints. The tension between the open-ended, process-oriented nature of adversarial cooperation described above and the pragmatic demands of decision-making is a real one, and navigating it effectively will—at least on some occasions—be essential to success. On one level, this tension reflects a fundamental dilemma of any moral engagement in a pluralistic democracy. However, this does not obviate the need for legitimate and justified decision-making. There is an inherent trade-off between the inclusivity and epistemic humility required to engage different perspectives authentically, and the closure and decisiveness needed to translate moral deliberation into collective action. If openness is prioritised too heavily, there is the risk of a kind of moral paralysis or relativism that fails to resolve pressing issues. But if there is a rush to decide and enforce uniform policies this creates a risk of marginalising dissenting voices and fracturing social cohesion.

Adversarial cooperation offers some strategies for managing this dilemma, but they are not panaceas. The techniques outlined above can help to surface underlying interests and generate creative solutions that integrate diverse concerns. They may also help to legitimise and channel moral contestation in productive directions. And methods from the arts and humanities can foster the empathy and mutual understanding needed to maintain social cohesion amidst ongoing disagreement. But ultimately, practising adversarial cooperation in the real world will require all involved to exercise judgement and balance competing considerations in context-dependent ways. Striking the right balance between openness and resolution in value-based decision-making will require an ongoing process of experimentation, learning and adaptation. It will depend on cultivating a democratic ethic that values both moral pluralism and the need for cooperative action in the face of pressing challenges. This is not a simple or one-time task, but an enduring responsibility as our social and technological contexts evolve. Adversarial cooperation offers a promising framework for this effort, but realising its potential will require sustained creativity and commitment from bioethicists, policymakers, and citizens alike.

In the development and refining of adversarial cooperation approaches it will also be essential to engage deeply with the practical wisdom— the ‘moral craft’—of those on the frontlines of ethical decision-making.^32^ By learning from different experiences and perspectives, we can hope to craft strategies that are both principled and pragmatic—that honour the depth of our moral differences while still enabling us to act together in pursuit of the common good.

## The implications of adversarial cooperation for rethinking bioethics

Adversarial cooperation places particular value on epistemic humility, flexibility and the ability to navigate uncertainty and disagreement constructively over time. This will require rethinking bioethics. If the central challenge of bioethics in a pluralistic society is not only to adjudicate between competing moral theories but also to navigate diverse lived moral experiences and worldviews, then a different kind of expertise may be required. Adversarial cooperation suggests that the most critical competencies for bioethicists may involve an interweaving of philosophical analysis and critical reflection with the ability to facilitate dialogue across difference, to synthesise insights from multiple disciplinary and experiential perspectives and to craft provisional solutions that can command broad legitimacy in the face of persistent disagreement.

This implies a model of bioethical expertise that is collaborative, contextual and transdisciplinary. It suggests that bioethicists need to be skilled not just in ethical reasoning but in mediation, conflict resolution, narrative analysis and participatory decision-making. They need to be able to move fluidly between different disciplinary languages and modes of inquiry, and to cultivate the epistemic humility and cultural competence required to engage authentically with diverse communities. Importantly, this vision of bioethical expertise suggests that legitimate bioethical authority in a pluralistic context must be grounded in the ability to facilitate inclusive and trustworthy processes of moral deliberation and decision-making. This is a more dynamic and relational conception of ethics expertise, one that is earned through demonstrated commitments to transparency, accountability and responsiveness to diverse stakeholders. It implies that the credibility of bioethicists depends less on their ability to provide definitive answers than on their capacity to navigate complex moral terrain in ways that are both principled and pragmatic, and to foster a culture of reason-giving and mutual respect amidst difference. A vitally important feature of any bioethics adequate to the tasks outlined above is going to be a sophisticated and inclusive understanding of history and of historical analysis. Cross-disciplinary work forging sophisticated connections between bioethicists, historians and other humanities scholars will be of crucial importance.

Cultivating this kind of expertise and authority will require significant changes in the way bioethicists are trained, socialised and rewarded. It will demand a much greater emphasis on interdisciplinarity in bioethics education and practice, with a focus on developing skills in facilitation, communication and collaborative problem-solving alongside traditional philosophical competencies. It will require building more robust partnerships between bioethics and fields like conflict resolution, community organising and participatory research. It will also necessitate grappling with the power dynamics and structural inequities that shape whose voices and perspectives are heard in bioethical deliberation and working to create more inclusive and equitable spaces for moral discourse and decision-making.^v^ This will require bioethicists to be more reflexive about their own social locations and biases, and to actively seek out and amplify marginalised knowledges and experiences.

Ultimately, reimagining bioethical expertise and authority is about more than just expanding the methodological toolbox of bioethicists—though training in methods such as those described above will be crucial. It is about fundamentally reorienting the field towards a more collaborative, contextual and democratic vision of its role and responsibilities in a pluralistic society. It is about recognising that in a world of deep and persistent moral disagreement, the most vital contribution of bioethics will be its capacity to hold space for difficult questions, to navigate uncertainty and complexity with appropriate humility and to model the kind of inclusive and empathetic engagement across difference that is the foundation of cooperative moral life.^33^ ^34^

## Conclusion

Polarisation around conflicting values—particularly when combined with pervasive misunderstanding and disagreements about what constitutes legitimate expertise—poses important challenges to the achievement of the shared purpose and collective action necessary for addressing the most important problems facing the world today. It is also an important obstacle to the achievement of sustainable, peaceful pluralistic societies.

In this paper, I argue that while adversarial collaboration and antagonistic cooperation arise from distinct contexts, an integration of some of their insights, what I have called ‘adversarial cooperation’, enriched by arts and humanities perspectives, inclusive, self-critical engagement, and philosophical rigour offers a set of strategies capable of enabling bioethics to engage productively and respectfully with seemingly intractable differences of values, commitments and interests. After outlining some of the features of a structured adversarial cooperation process, I described four sets of challenges that any successful development and implementation of it would need to address: a range of ethical questions; tensions between commitments to substantive and procedural values; the problem of fake news and misinformation; and finally, the need to make decisions and develop policy. The paper concluded with some reflections on ways in which the preceding discussion of adversarial cooperation and strategies for its realisation suggest the need for a rethinking of many of the commitments, methods and core concepts of much contemporary bioethics and a greater emphasis on global health bioethics as a forum for a renewal of bioethics as a discipline.

## Data Availability

No data are available.

## Footnotes

An important feature of Kahneman’s uses of adversarial collaborations is that in the resulting papers, adversaries were given equal length within the manuscript to state their positions.

But see the section on problems of decision-making below.

https://blackbrownbioethics.blogs.bristol.ac.uk/
